# Aging and the Optimal Viewing Position Effect in Visual Word Recognition: Evidence From English

**DOI:** 10.1037/pag0000163

**Published:** 2017-04-13

**Authors:** Lin Li, Sha Li, Jingxin Wang, Victoria A. McGowan, Pingping Liu, Timothy R. Jordan, Kevin B. Paterson

**Affiliations:** 1 Academy of Psychology and Behavior, Tianjin Normal University; 2 Department of Neuroscience, Psychology and Behaviour, University of Leicester; 3 Centre on Aging Psychology, Institute of Psychology, Chinese Academy of Sciences; 4 Cognition and Neuroscience Research Laboratory, Department of Psychology, Zayed University Dubai; 5 Department of Neuroscience, Psychology and Behaviour, University of Leicester

**Keywords:** aging, visual word recognition, optimal viewing position

## Abstract

Words are recognized most efficiently by young adults when fixated at an optimal viewing position (OVP), which for English is between a word’s beginning and middle letters. How this OVP effect changes with age is unknown but may differ for older adults due to visual declines in later life. Accordingly, a lexical decision experiment was conducted in which short (5-letter) and long (9-letter) words were fixated at various letter positions. The older adults produced slower responses. But, crucially, effects of fixation location for each word-length did not differ substantially across age groups, indicating that OVP effects are preserved in older age.

Where a reader fixates initially within a word influences the efficiency with which the word can be identified (O’Regan, 1981; see also Dunn-Rankin, 1978; Grainger, Dufau, & Ziegler, 2016; Rayner, 1979). In particular, words read in isolation are recognized quickest and most accurately when initially fixated at an optimal viewing position (OVP). The location of the OVP varies across different writing systems (e.g., Deutsch & Rayner, 1999; Jordan, Almabruk, McGowan, & Paterson, 2011; Liu & Li, 2013; Liu, Liu, Han, & Paterson, 2015). But for Latinate languages (e.g., English, French) it is between a word’s beginning and middle letters, although the exact location varies with word length.

The advantage of fixating this location in words read in isolation is shown clearly in studies that have examined lexical decisions or naming times for words, which are faster for words fixated at the OVP than other letter locations (e.g., Farid & Grainger, 1996; Hyönä & Bertram, 2011; Jordan, Paterson, Kurtev, & Xu, 2010; O’Regan, 1981; O’Regan & Jacobs, 1992; O’Regan, Lévy-Schoen, Pynte, & Brugaillère, 1984; Vitu, O’Regan, & Mittau, 1990). These effects of fixation location are not modulated by linguistic factors such as the lexical frequency of words and so appear to affect a prelexical stage of processing (e.g., Hyönä & Bertram, 2011; O’Regan & Lévy-Schoen, 1987). Moreover, studies that have examined the influence of initial fixation location on eye movement behavior for isolated words show that initial fixations are longer and the probability of a refixation lower for words initially fixated at the OVP than other letter locations (e.g., O’Regan & Lévy-Schoen, 1987; Vitu, McConkie, Kerr, & O’Regan, 2001). This trade-off between fixation duration and refixation probability reveals that fixating a suboptimal location in a word can rapidly trigger an eye movement and is attributed to readers terminating a fixation at an unfavorable location to refixate the word at a more favorable location.

A full explanation for the OVP effect remains elusive (see Brysbaert & Nazir, 2005; Stevens & Grainger, 2003). However, it seems clear that the rapid drop-off in acuity that occurs with increasing eccentricity from the center of gaze (e.g., Anstis, 1974; Hilz & Cavonius, 1974) is an especially important component. Indeed, while linguistic information is acquired from beyond the fixated word during textual reading (e.g., McConkie & Rayner, 1975; see also Jordan, McGowan, Kurtev, & Paterson, 2016; Jordan, McGowan, & Paterson, 2013), much of this input is low resolution and used to guide forward-directed saccades and parafoveally preprocess upcoming words.^1^ By contrast, high resolution input is acquired from only a narrow region at the center of gaze so that as few as 4–5 letters can be identified accurately (i.e., >80% correct) on each glance (e.g., O’Regan, Lévy-Schoen, & Jacobs, 1983; Yu, Legge, Wagoner, & Chung, 2014). Consequently, when a word is encountered in isolation (and its recognition cannot benefit from context or parafoveal preprocessing), there is a perceptual advantage to fixating the word at its very center, to maximize the number of letters that project to high-acuity retinal regions (e.g., O’Regan, 1981). However, this perceptual advantage is thought to be modulated by other factors, including perceptual learning (Nazir, Ben-Boutayab, Decoppet, Deutsch, & Frost, 2004), the distribution of linguistic information within words (Farid & Grainger, 1996; Jordan et al., 2011; O’Regan, 1981; O’Regan et al., 1984) and hemispheric asymmetries in the processing of words (Brysbaert & Nazir, 2005), so that the actual location of the OVP is shifted to the left of the center of words.

Crucially, however, while effects of fixation location on isolated word recognition have been investigated extensively for young adults, the influence of normal aging on these effects is unclear. In particular, because of visual declines that occur naturally in later life (see Owsley, 2011), older adults have lower acuity and reduced sensitivity to fine visual detail compared with young adults (e.g., Elliot, Yang, & Whitaker, 1995; see also Jordan, McGowan, & Paterson, 2014), and these reductions are greater further from the center of gaze (e.g., Collins, Brown, & Bowman, 1989; Crassini, Brown, & Bowman, 1988). Older adults may therefore experience a steeper decline in visual sensitivity with increasing retinal eccentricity. Consequently, while fixating a word’s OVP may benefit the recognition of isolated words by both young and older adults, costs associated with fixating suboptimal locations in words may be greater for older than younger adults due to this decline in visual sensitivity. Moreover, this may be an important component of the greater word recognition difficulty that older adults experience (e.g., Allen, Madden, Weber, & Groth, 1993; Cohen-Shikora & Balota, 2016; Ratcliff, Thapar, Gomez, & McKoon, 2004). Age differences in effects of fixation location may also be greater for longer words, as fixating a longer word at a suboptimal location will cause a larger proportion of that word’s letters to project to lower acuity retinal regions. But while studies show that older adults are slower to recognize words, the contribution of fixation location to this slower word recognition remains to be determined.

Accordingly, to address this issue, the present experiment investigated the effect of fixation location on the recognition of short (5-letter) and long (9-letter) English words (and pseudowords) by young (18–25 years) and older (65+ years) adults. Following previous research with young adults, the present study assessed word recognition using the lexical-decision task. Stimuli were presented to participants at different locations straddling a central fixation cue so that words (and pseudowords) were first fixated at beginning, middle, or end letter locations. Instructions alone have been shown to be ineffective for achieving fixation accuracy (Jordan, Patching, & Milner, 1998; Jordan et al., 2010; also Jordan & Paterson, 2009), so an eye-tracker was used to ensure participants fixated the desired location prior to each stimulus presentation and to assess the location and duration of fixations on words.

Previous research suggests that lexical decisions are slower but not less accurate for older adults (e.g., Ratcliff et al., 2004), and so we expected to observe similar overall effects of age on the accuracy and latency of lexical decisions in the present experiment. Studies also show that lexical decisions by young adult readers of Latinate languages are fastest for words fixated between their beginning and middle letters (O’Regan & Jacobs, 1992; see also Jordan et al., 2010). We therefore expected to replicate these effects of fixation location for the young adults in the present study. But the findings were likely to reveal if word recognition by older adults shows similar sensitivity to the location of initial fixations in words, so that older adults also benefit from fixating a word at its OVP. Moreover, if fixating a word at a suboptimal letter location is more costly to word recognition for older than younger adults, we should observe an interaction between age-group and fixation location such that the slowdown in responses times produced by fixating words at suboptimal locations is larger for the older adults. Moreover, as a larger proportion of letters in longer words will project to lower acuity retinal regions when words are fixated at a suboptimal location, the 9-letter words may produce clearer OVP effects than the 5-letter words (e.g., O’Regan & Jacobs, 1992), and also reveal clearer age differences in these effects.

It was of further concern to establish the influence of initial fixation location on eye movement behavior for words read in isolation differs for young and older adults. Previous research with young adults shows that initial fixations are longer and the probability of a refixation lower when isolated words are first fixated at the OVP than other letter locations (e.g., O’Regan & Lévy-Schoen, 1987; Vitu et al., 2001). We expected to replicate these effects for the young adults in the present study. Fixations were also likely to be longer or refixation probabilities higher for the older than younger adults if, as in textual reading, older adults require longer to visually process words (e.g., Rayner, Reichle, Stroud, Williams, & Pollatsek, 2006). Crucially, however, the findings would reveal if the eye movements of young and older adults show similar sensitivity to the location of initial fixations. Indeed, if the eye movements of older adults are as responsive to the location of fixations in words as those of young adults, initial fixations should be longer and refixation probabilities lower for both age groups when words are first fixated at the OVP. By contrast, an interaction between age-group and fixation location in fixation duration or refixation probability might indicate differences in the eye movement behavior of the young and older adults. Finally, we examined the location of refixations in words to establish if refixations are targeted toward the word’s OVP.

## Method

This research was conducted with the approval of the Research Ethics Committee in the School of Psychology at the University of Leicester, and in accordance with the principles expressed in the Declaration of Helsinki. All participants gave informed consent in writing.

### Participants

Participants were 15 young adults (*M* = 22 years, range = 17–25 years) from the University of Leicester and 15 older adults (*M* = 75 years, range = 65–82 years) from the local community. All participants were native English readers, and young and older adults were matched for years of formal education (young adults, *M* = 14 years, range = 13–15 years; older adults, *M* = 15 years, range = 12–20 years, *t* < 1.4). OVP effects may vary as a function of hemispheric language dominance (e.g., Hunter & Brysbaert, 2008). Participants were therefore selected for right-handedness, and so typical (left hemisphere) dominance, using the revised Annett Handedness Questionnaire (Annett, 1970). Participants were also screened for near-distance (40 cm) acuity within the normal range (better than 20/40 in Snellen values), and to ensure they were correctly refracted at the viewing distance in the experiment, using a high-contrast Early Treatment Diabetic Retinopathy Study (ETDRS) letter chart (Ferris & Bailey, 1996). The older adults had poorer acuity than the young adults (older adults, *M* = 20/30; young adults, *M* = 20/20, *t*(28) = 5.05, *p* < .001), as is typical for these age groups (Elliot et al., 1995). Finally, older adults were screened for normal cognitive abilities using the Montreal Cognitive Assessment (Nasreddine et al., 2005).

### Stimuli and Design

Stimuli were 60 short (5-letter) and 100 long (9-letter) nouns selected to have medium lexical frequency according to both the CELEX database (Baayen, Piepenbrock, & Gulikers, 1995) and SUBTLEX-UK database (van Heuven, Mandera, Keuleers, & Brysbaert, 2014). The short and long words were closely matched for lexical frequency (CELEX counts per million: short words, *M* = 37.8; long words, *M* = 40.0; *t* < 1; SUBTLEX-UK log frequency counts: short words, *M* = 4.3; long words, *M* = 4.2; *t* < 1.3). Another 60 short and 100 long nouns selected to have medium lexical frequency were used to create pseudoword stimuli by the transposition of two interior letters. Each stimulus was displayed to participants so that they initially fixated a specific letter position (letter positions 1, 3, and 5 in short words/pseudowords, and 1, 3, 5, 7, and 9 in long words/pseudowords). Stimuli were sampled pseudorandomly for presentation at each fixation location so that each participant viewed a different 20 short words and pseudowords, and a different 20 long words and pseudowords, at each letter position. Across each age-group, participants viewed each short and long word an equal number of times at each letter position. Only the data for words were assessed and analyses were computed separately for the short and long words. Accordingly, the experiment had a mixed design for each word length, with the between-participants factor age-group (young adult, older adult) and within-participants factor fixation location (letter positions 1, 3, and 5 in short words, and 1, 3, 5, 7, and 9 in long words).

### Apparatus and Procedure

**Figure 1 fig1:**
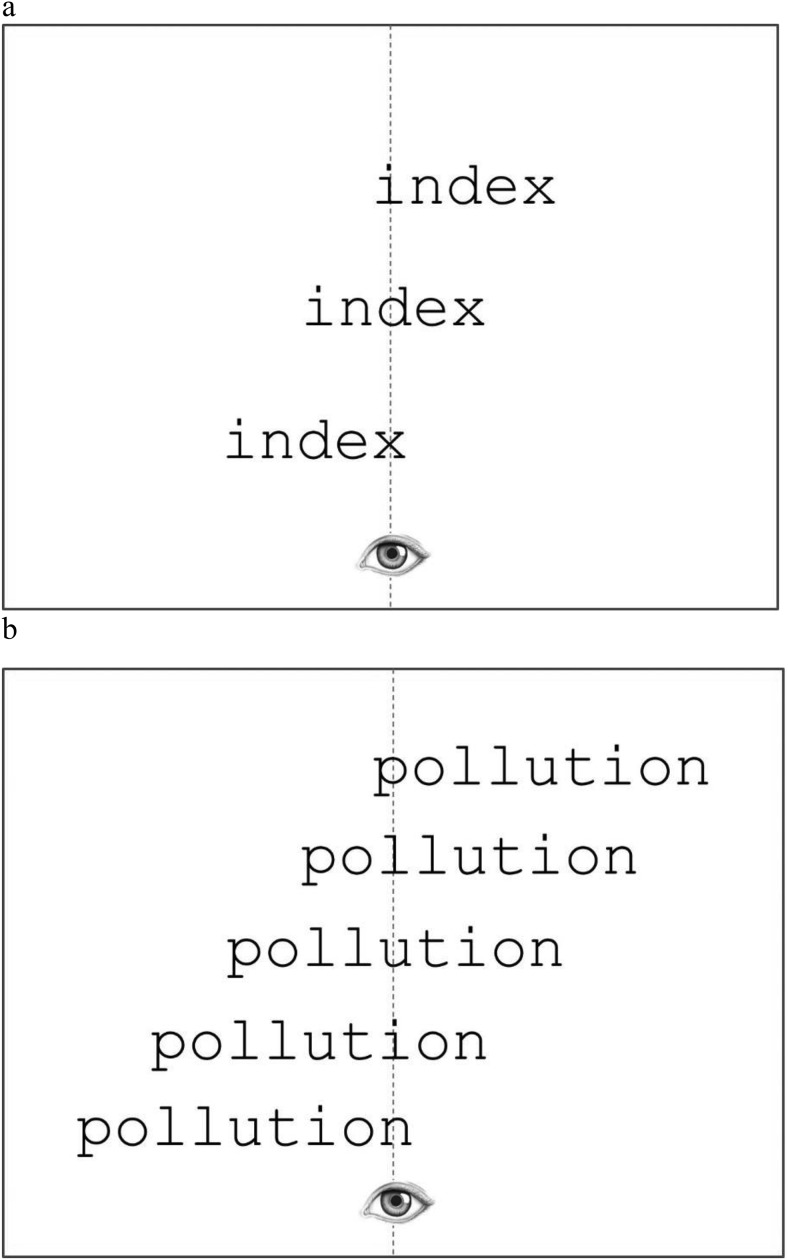
Example of initial fixation locations in (a) short and (b) long words.

Stimuli were presented on a 21-inch high-resolution ViewSonic CRT monitor (with a 100 Hz refresh rate) at a viewing distance of 80 cm. At this distance, 4 letters subtended approximately 1.2° horizontally, and so stimuli were of normal size for reading (Rayner & Pollatsek, 1989). Participants were instructed to fixate a designated fixation point (a small dot) at the center of the screen at the start of each trial. An EyeLink 1,000 eye tracker (SR Research) was used to ensure accurate fixation of this designated fixation location prior to each stimulus presentation and to record the location and duration of fixations. The eye-tracker has high spatial (.01°) and temporal resolution (1,000 Hz). A 3-point horizontal calibration procedure ensured all participants were calibrated with accuracy <.30°, which is typical for eye movement experiments. Participants had to fixate within a window approximately .30° wide centered on the fixation dot to initiate each stimulus presentation. Once the participant fixated within this region for 50 ms, the fixation dot was replaced by a stimulus, presented briefly (for 150 ms). This was shown at different offsets relative to the fixation dot, so that initial fixations were made at beginning, middle, or end letter positions (see Figure 1). Participants were instructed to make a lexical decision as quickly and accurately as possible for each stimulus presentation by pressing one of two keys on a response pad using their right hand. Each participant viewed a total of 160 words and 160 pseudowords along with 8 practice words and pseudowords presented at the start of the experiment. The experiment lasted approximately 30 min for each participant.[Fig-anchor fig1]

## Results

**Figure 2 fig2:**
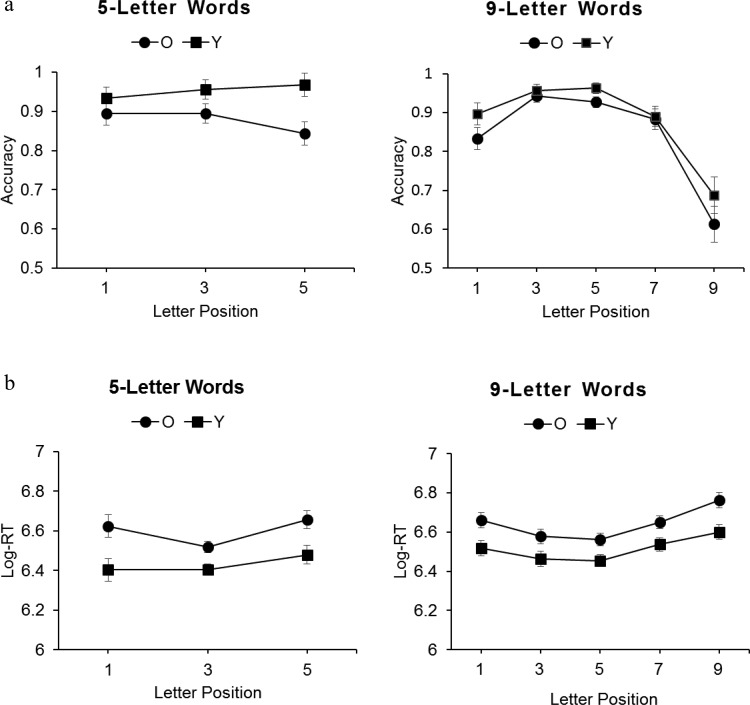
Young and older adults’ (a) mean lexical decision accuracy and (b) log-transformed response latencies (ms). Error bars represent the standard error of the mean.

**Figure 3 fig3:**
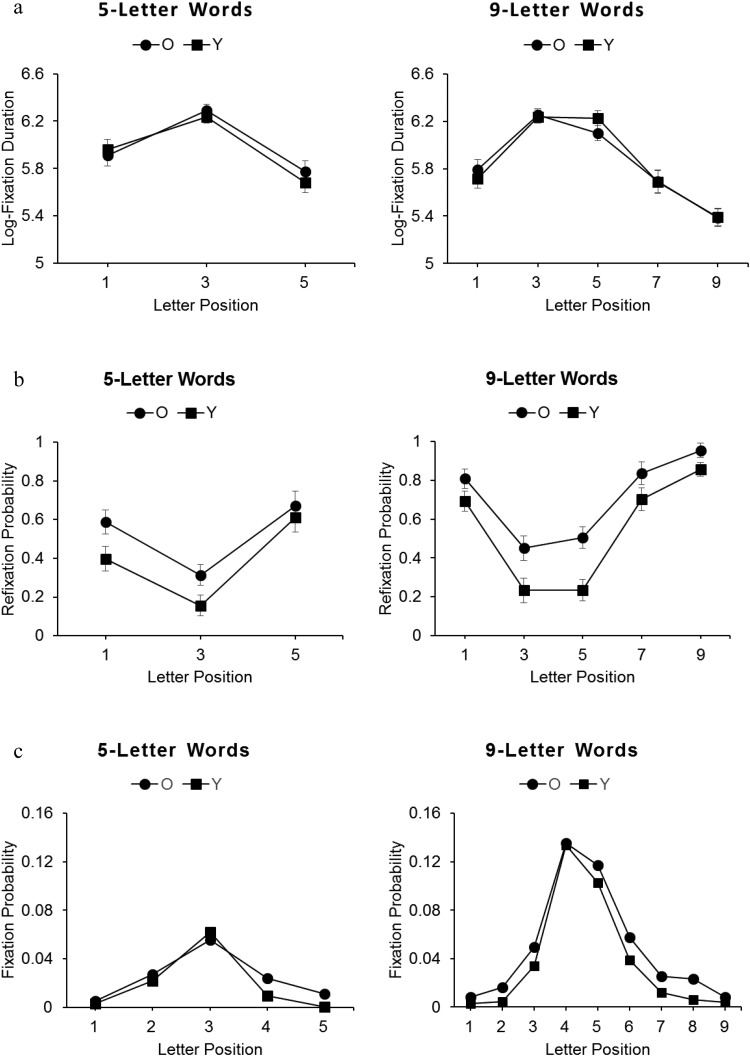
Young and older adults’ (a) log-transformed initial fixation durations (ms), (b) refixation probabilities, and (c) distribution of refixation locations, for short and long words. Error bars represent the standard error of the mean.

Response accuracy for lexical decisions was high for all participants (>85%) indicating that both young and older adults recognized words effectively. Data were analyzed for word presentations only. Reaction time and fixation duration data were log-transformed, although transformed and untransformed data showed the same patterns of effects. Response accuracy and log-transformed response times, log-transformed initial fixation durations, and refixation probabilities for words receiving a correct lexical decision, were analyzed separately for the short and long words using a mixed-design analysis of variance (ANOVA) with the between-participants factor age-group (young adults, older adults) and within-participants factor fixation location (letter positions 1, 3, and 5 in short words, and 1, 3, 5, 7, and 9 in long words). Error variance was computed separately across participants (*F*_1_) and stimuli (*F*_2_). Pairwise comparisons were Bonferroni-corrected. Figure 2 shows mean accuracy and Response Times (RTs) and Figure 3 mean initial fixation durations, refixation probabilities, and distribution of the location of refixations in words for both age groups.[Fig-anchor fig2][Fig-anchor fig3]

In addition to traditional ANOVA analyses, Bayes factors (Kass & Raftery, 1995) were computed for each measure to assess the strength of evidence for null and alternative hypotheses for key comparisons. These computations were performed using the Bayes Factor package (version 0.9.12–2; Rouder, Morey, Speckman, & Province, 2012) in R (R Development Core Team, 2016). Marginal likelihood was obtained using Monte Carlo sampling, with iterations set at 100,000 and the scaling factor for g-priors set to 0.5 (see Abbott & Staub, 2015). Both participants and stimuli were specified as random variables for reported analyses, although the same patterns of results were obtained when analyses were performed with only participants or stimuli specified as a random variable. Model comparisons were computed initially with the null model as the default denominator model, and alternative models directly compared with each other by computing the ratio of their Bayes factors (following Abbott & Staub, 2015). However, the same pattern of effects was obtained when comparisons were made between alternative models by specifying the most strongly preferred model as the denominator model in analyses. Model comparisons were made using interpretation categories set out by Vandekerckhove, Matzke, and Wagenmakers (2014), so that Bayes factors (BFs) > 3 were taken to provide weak to moderate support for a model over an alternative model (including over the null model during initial model comparisons), and BFs > 10 to provide strong support, while BFs < 1 were taken to provide evidence against a model and in favor of the alternative (or null) model.

### Response Accuracy

For the short words, there was a main effect of age group, *F*_1_(1, 28) = 4.86, *p* < .05, η_p_^2^ = .15, and *F*_2_(1, 59) = 15.23, *p* < .001, η_p_^2^ = .21, due to more accurate responses by the young (95%) than older (87%) adults. The main effect of fixation location and interaction between age group and fixation location were not significant, *F* values < 3, and so response accuracy did not vary as a function of fixation location for either age-group.

For the long words, there was a main effect of age group by stimuli only, *F*_1_(1, 28) = 2.41, *p* > .05, η_p_^2^ = .08, and *F*_2_(1, 99) = 7.48, *p* < .01, η_p_^2^ = .07, and a main effect of fixation location, *F*_1_(4, 112) = 47.53, *p* < .001, η_p_^2^ = .63, and *F*_2_(4, 396) = 71.04, *p* < .001, η_p_^2^ = .42, but no interaction, *F* values < 1.6. Accuracy was equally highest for fixations at letter positions 3 (95%) and 5 (95%), equally lower for positions 1 (86%) and 7 (89%), and lowest at position 9 (65%). Thus, lexical decisions by both age groups were most accurate for long words fixated between their beginning and middle letters.

Compared with a null model, Bayes factors for the short words provided strongest support for a model containing only a main effect of age group (BF = 1,746), and weaker support for models containing additive (BF = 23) or interactive (BF = 3) effects of age group and fixation location. As the question of interest concerned whether the data are best accounted for by a model that includes an interactive effect of age group and fixation location, we directly compared the interactive model against the preferred model by calculating the ratio of their BFs. The BF for this comparison was 532, strongly favoring a model containing only an effect of age group. Bayes factors for response accuracy for the short words therefore provided strong support for the ANOVA and evidence against an interactive effect of age group and fixation location.

BFs for the long words were high compared with the null model for models that included either additive effects (BF = 6.4 × 10^63^) or interactive effects (BF = 2.5 × 10^61^) of age group and fixation location, although model comparison strongly favored the additive model (BF = 256). Bayes factors for response accuracy for the long words therefore also provided support for the ANOVA findings and evidence against an interactive effect.

### Reaction Times

For the short words, there was a main effect of age group, *F*_1_(1, 28) = 11.31, *p* < .01, η_p_^2^ = .29, and *F*_2_(1, 59) = 47.06, *p* < .001, η_p_^2^ = .44, due to slower responses by the older than younger adults. There was also a main effect of fixation location, *F*_1_(2, 56) = 4.87, *p* < .05, η_p_^2^ = .15, and *F*_2_(2, 118) = 5.20, *p* < .01, η_p_^2^ = .08, but no interaction, *F* values < 1.3. Reaction times for both age groups were shortest for letter positions 1 and 3 and longest at position 5. Young and older adults therefore showed a similar response time benefit for fixating a region encompassing the beginning and middle letters of short words.

For the long words, there was a main effect of age group, *F*_1_(1, 28) = 10.11, *p* < .01, η_p_^2^ = .27, and *F*_2_(1, 87) = 100.47, *p* < .001, η_p_^2^ = .54, due to slower responses by the older than younger adults. There was also a main effect of fixation location, *F*_1_(4, 112) = 16.48, *p* < .001, η_p_^2^ = .37, and *F*_2_(4, 348) = 16.77, *p* < .001, η_p_^2^ = .16, but no interaction, *F* values < 1. Response times for both age groups were equally fastest for letter positions 3 and 5, equally slower at positions 1 and 7, and slowest at position 9. Both age groups therefore showed a response time benefit of initially fixating between the beginning and middle letters of longer words.

Bayes factors analyses produced high BFs for models containing additive and interactive effects over null models for both short word (additive model, BF = 7.5 × 10^7^; interactive model, BF = 3.3 × 10^5^) and long words (additive model, BF = 4.8 × 10^21^; interactive model, BF = 5.3 × 10^18^). Model comparisons showed that additive models were strongly favored over interactive models for both the short words (BF = 227) and the long words (BF = 906), confirming the ANOVA findings and providing evidence for an additive rather interactive influence of age group and fixation location.

### Initial Fixation Duration

For the short words, there was a main effect of fixation location, *F*_1_(2, 56) = 38.87, *p* < .001, η_p_^2^ = .58, and *F*_2_(2, 118) = 79.66, *p* < .001, η_p_^2^ = .58, but no main effect of age group or an interaction, *F* values < 1. Fixations were longest for letter position 3, shorter for position 1, and shortest for position 5. For the long words, there was a main effect of fixation location, *F*_1_(4, 112) = 81.58, *p* < .001, η_p_^2^ = .74, and *F*_2_(4, 348) = 81.92, *p* < .001, η_p_^2^ = .51, but no main effect of age group or an interaction, *F* values < 1. Fixations for young and older adults were equally longest at letter positions 3 and 5, equally shorter at positions 1 and 7, and shortest at position 9. Similarly to previous studies, initial fixations by the young adults were longest at the OVP of both short and long words, and this influence of fixation location on initial fixation duration was essentially the same for the older adults.

Bayes factors analyses produced high BFs for models containing additive and interactive effects of age group and fixation location over null models for both short words (additive model, BF = 4.0 × 10^26^; interactive model, BF = 2.9 × 10^25^) and long words (additive model, BF = 6.2 × 10^153^; interactive model, BF = 8.8 × 10^152^). Model comparisons showed that additive models were favored over interactive models for short (BF = 14) and long (BF = 7) words, confirming ANOVA findings and providing evidence favoring an additive rather interactive influence of age group and fixation location.

### Refixation Probabilities

For the short words, there was a main effect of fixation location, *F*_1_(2, 56) = 32.23, *p* < .001, η_p_^2^ = .54, and *F*_2_(2, 118) = 53.03, *p* < .001, η_p_^2^ = .47, a main effect of age group by stimuli only, *F*_1_(2, 56) = 3.78, *p* > .05, η_p_^2^ = .12, and *F*_2_(1, 59) = 23.70, *p* < .001, η_p_^2^ = .29, and no interaction, *F* values < 1. Fixations at letter position 3 produced the lowest refixation rates, and those at positions 1 and 5 produced equally higher rates.

For the long words, there was a main effect of fixation location, *F*_1_(4, 112) = 73.69, *p* < .001, η_p_^2^ = .72, and *F*_2_(4, 348) = 123.58, *p* < .001, η_p_^2^ = .59, and a main effect of age group, *F*_1_(1, 28) = 9.67, *p* < .01, η_p_^2^ = .26, and *F*_2_(2, 87) = 73.20, *p* < .001, η_p_^2^ = .46, but no interaction, *F* values < 1. Refixation probabilities were equally lowest following initial fixations at letter positions 3 and 5, equally higher at positions 1 and 7, and highest at position 9. In addition, the older adults had higher refixation rates than the young adults. The present findings therefore replicate previous findings for young adults showing that refixation rates are lower following an initial fixation at the OVP than other letter locations (e.g., O’Regan & Lévy-Schoen, 1987; Vitu et al., 2001). The findings additionally show that this effect is essentially the same for young and older adults but that older adults have generally higher refixation rates, consistent with their slower processing of words.

Bayes factors analyses produced high BFs for models containing additive and interactive effects for short words (additive model, BF = 1.8 × 10^21^; interactive model, BF = 1.5 × 10^20^) and long words (additive model, BF = 3.2 × 10^132^; interactive model, BF = 1.5 × 10^133^). Model comparisons showed that the additive model was favored over the interactive model for short words (BF = 12), but that the interactive model was favored for long words (BF = 5). The analyses for short words therefore support the ANOVA findings and provide evidence for an additive over an interactive influence of age group and fixation location for these words. But the analyses for long words revealed support for an interactive model that was not apparent in the ANOVA findings. This demonstrates that a rejection of the null based on *p* < .05 may occur even when Bayes factors reveal weak evidence against or even evidence in favor of the null (Rouder & Morey, 2011). In the present case, effects of fixation location on refixation probabilities were broadly similar across age groups, although suboptimal fixations produced a larger increase in refixation probabilities for young than older adults. This was most likely because fixations at optimal locations were already associated with higher baseline refixation probabilities for the older adults and so fixations at suboptimal locations had less scope to increase refixation probabilities for these readers.

### Refixation Locations in Words

Refixations tended to land at the center of short words and slightly to the left of the center of long words for both the young and older adults, indicating that refixations by both age groups were directed toward the OVP.

## Discussion

The present results showed clear effects of fixation location on isolated word recognition by young and older adults (confirmed by both traditional ANOVA analyses and analyses based on Bayes factors). For both age groups, lexical decisions were fastest for short (5-letter) and long (9-letter) words fixated initially between their beginning and middle letters. There was also an effect of fixation location on the accuracy of lexical decisions for the long words, such that accuracy was highest for both age groups for words fixated initially between their beginning and middle letters. No such effects were observed for the short words, most likely because recognition accuracy for these words was at ceiling for both age groups. The indication, therefore, is that young and older adults produce OVP effects for words read in isolation, so that words are recognized most efficiently when initially fixated between their beginning and middle letters, although the precise location of the OVP varies with word length.

These effects of fixation location were in line with findings for young adults in previous research (e.g., O’Regan & Jacobs, 1992) and provided novel evidence of effects of fixation location for older adults. However, unlike many previous studies, the present experiment used an eye-tracker to ensure participants accurately fixated the desired locations in words (see Jordan et al., 1998, 2010; Jordan & Paterson, 2009), and to avoid the contamination of effects by age differences in fixation control (see Kosnik, Fikre, & Sekuler, 1986). The older adults also made slower responses than young adults in the present experiment, consistent with findings from numerous other studies showing that older adults are slower to make lexical decisions (e.g., Allen et al., 1993; Cohen-Shikora & Balota, 2016; Ratcliff et al., 2004). Crucially, however, effects of fixation location on the accuracy and latency of lexical decisions did not differ substantially for the young and older adults (confirmed by Bayes factor analyses). Instead, the considerable similarity in the effects of fixation location on word recognition across the two age groups suggests young and older adults obtained similar benefits from initially fixating words at their OVP, and a similar cost when initially fixating other letter locations. Indeed, initially fixating a word at a suboptimal letter location was no more costly for the older than younger adults, and so there was no indication that age differences in the effects of fixation location are an important component of the greater word recognition difficulty that older adults experience.

The present findings also shed fresh light on the influence of fixation location on subsequent eye movement behavior for words read in isolation. In particular, fixations were longest and refixation probabilities lowest for both age groups for short words initially fixated at their center and long words initially fixated between their beginning and middle letters. These influences of fixation location on eye movements resonate with effects of OVP on word recognition in the present study, and are consistent with effects of fixation location on the eye movement behavior of young adults in other studies of isolated word recognition (e.g., O’Regan & Lévy-Schoen, 1987; Vitu et al., 2001). Such findings are often taken to show that readers rapidly terminate fixations at unfavorable locations to make a corrective eye movement to refixate words at more favorable locations. Consistent with this account, the present research showed also that refixations tend to be made at the center of short words and between the beginning and middle letters of long words, and so appear to be targeted toward the OVP.

Older adults also had higher refixation probabilities than young adults, consistent with other indications that older readers acquire word information more slowly (e.g., Rayner et al., 2006). However, whereas the traditional ANOVA analyses provided no indication of an interaction between age group and fixation location, Bayes factors indicated that such an interaction was likely for refixation probabilities for the long words (but not short words). Crucially, the general pattern of effects of fixation location on refixation probabilities for long words was similar for the young and older adults, although fixating a suboptimal rather than option location in these words produced a greater rise in refixation probability for the young adults than the older adults. This effect is most likely because the probability of a refixation was generally higher for the older adults even when their initial fixations landed at optimal locations in words, so that there was less scope for suboptimal fixations to increase refixation probability for these readers. Accordingly, the interaction effect did not reveal an important qualitative difference between young and older adults and the more general indication from the present findings is that fixation location exerts a rapid and broadly similar influence on the eye movements of young and older adults during isolated word recognition.

The correspondence between these effects of fixation location on isolated word recognition and effects in textual reading need to be investigated more fully. Other studies show that young and older adults exhibit a similar tendency to initially fixate words at a preferred viewing position, typically a little to the left of the center of words, during textual reading (Paterson, McGowan, & Jordan, 2013a; Rayner et al., 2006; see also Rayner, 1979). Such findings have been taken to show that, while many aspects of oculomotor function change with age (e.g., Irving, Steinbach, Lillakas, Babu, & Hutchings, 2006), eye movement control during reading is resistant to effects of normal aging, and the present findings resonate with this view. It will be important, however, for future research to establish if, in line with the present findings for isolated words, the location of initial fixations on words during textual reading affects processes of word recognition and subsequent eye movement behavior similarly for young and older adults. For instance, it is unclear if fixating a suboptimal location in words during textual reading produces disruption to word recognition and an increase in refixation probabilities for young and older adults similar to that observed for isolated words in the present study. It will also be important to gain a broader understanding of how older readers adapt to changes in visual, attentional, and memory capabilities that occur naturally with older age (see Gordon, Lowder, & Hoedemaker, 2016, for a recent review). For instance, older readers may compensate for their poorer visual processing of text by relying more heavily on coarse-scale visual cues to word identities (Jordan et al., 2014; Paterson, McGowan, & Jordan, 2013b), making greater use of context (see Rayner et al., 2006) or drawing upon their greater knowledge and experience of words (Payne, Gao, Noh, Anderson, & Stine-Morrow, 2012; Verhaeghen, 2003), although how these factors might compensate for age-related visual and cognitive declines remains to be determined.

In sum, the present findings reveal striking similarities in the influence of fixation location on word recognition and eye movement behavior by young and older adults. In particular, the findings show that the advantage for word recognition of initially fixating the OVP in words, and the rapid use of eye movements to correct for suboptimal fixation locations, are highly resilient and appear to be preserved in older age.

## Footnotes

1 Words can be recognized when presented in peripheral vision (for overviews, see e.g., Bradshaw & Nettleton, 1983; Chiarello, 1988; Jordan & Paterson, 2009), even by older adults (Grabbe & Allen, 2013; Yu, Cheung, Legge, & Chung, 2010). However, performance is typically much slower and less accurate compared with word recognition in central vision, due to reductions in acuity and sensitivity to visual detail (for a review, see Owsley, 2011), as well as increased effects of visual crowding (i.e., difficulty recognizing visual objects, such as letters, when closely surrounded by similar objects; see e.g., Bouma, 1970; Levi, 2008) with increased retinal eccentricity.
